# Neglected lunate dislocation presenting as carpal tunnel syndrome

**DOI:** 10.3109/23320885.2014.993397

**Published:** 2015-01-06

**Authors:** Eren Cansü, Ahmed Majid Heydar, Anar Elekberov, Mehmet Bekir Ünal

**Affiliations:** ^a^ Marmara University Medical School, Department of Orthopedic and Traumatology, Istanbul, Turkey; ^b^ Medipol University, Department of Orthopedic and Traumatology, Istanbul, Turkeyz

**Keywords:** Etiology, hand, lunate dislocation, median nerve, syndrome, trauma, unilateral carpal tunnel syndrome

## Abstract

Most of carpal tunnel syndrome cases are idiopathic, and secondary causes are so rare that can be easily missed. We present a patient with neglected undiagnosed lunate dislocation compressing on median nerve causing its signs and symptoms.

## Introduction

Carpal tunnel syndrome (CTS) is median nerve entrapment neuropathy due to compression of the median nerve in the carpal tunnel, which is a cylindrical passage connecting the volar forearm with the palm. The carpal tunnel is bounded by the transverse arch of the carpal bones dorsally and by the flexor retinaculum anteriorly. Typical symptoms of median nerve compression are tingling and numbness in the radial three-and-half digits with deep and diffuse pain in the hand, radiating proximally to the forearm and nocturnally alleviated. Functional disturbances resulting from compression lesions range all the way from loss of some types of sensation and muscle power to total sensory loss and muscle atrophy if the causative factor is not eliminated.

CTS is by far the most common peripheral entrapment neuropathy. Middle age, female gender, obesity, and gravidity are risk factors. Any factors which can decrease the size of the tunnel, such as abnormality of carpal bones and flexion or extension posture of the wrist, are thought to be the cause of CTS. However, in the most cases the etiology is unknown.

The diagnosis can be made by demonstrating a history of the clinical features described above, as well as clinical examination. Tinel’s test, Phalen’s test, compression test, and atrophy of the thenar muscles are of great value in making a diagnosis. Electrodiagnostic studies such as nerve conduction velocity and electromyogram (EMG) are confirmatory tests; they are also helpful in the diagnosis of other types of nerve entrapment in the upper limb and for postoperative evaluation. Computerized tomography (CT) scanning, ultrasound, and magnetic resonance imaging can all be used in detecting any abnormality within the carpal canal.

The treatment depends on whether the CTS is idiopathic or due to identifiable cause. In idiopathic patients with mild symptoms, and when there is no sign of thenar atrophy, night splint and steroid injection can be attempted. In other cases who failed conservative treatment, the treatment should be directed toward decompression of the carpal tunnel and division of the deep transverse carpal ligament either by open or endoscopic approaches. However, such treatments might not be effective in patients with secondary causes, misdiagnosed as idiopathic CTS, so the treatment should be directed toward underlying cause.

## Case report

**Figure 1.**
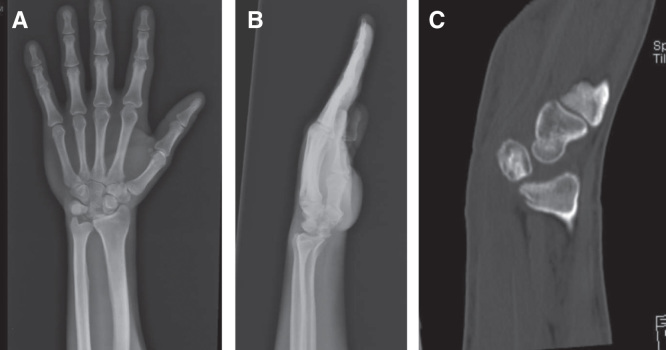
**Preoperative X-rays (*a* and *b*) and CT scan (*c*) showing volar dislocation of the lunate bone.**

**Figure 2.**
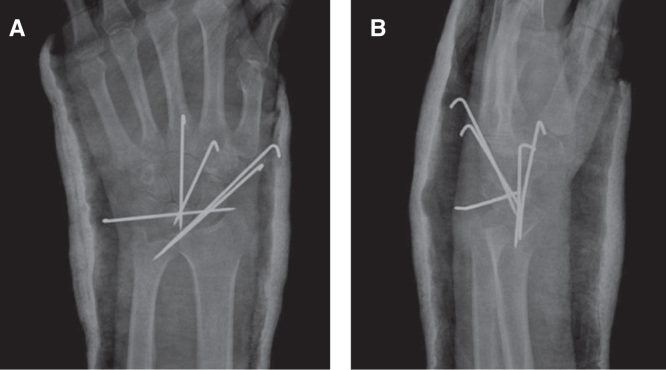
**Immediate postoperative X-rays (*a* and *b*) are shown.**

**Figure 3.**
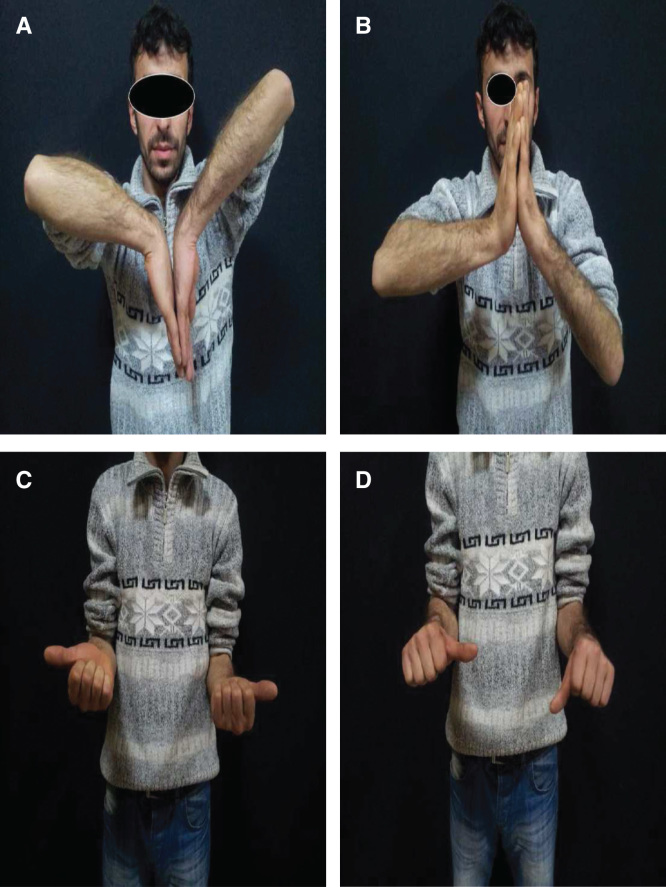
**Clinical photos showing the functional results of the patients at second year postoperatively (*a*, *b*, *c*, and *d*).**

**Figure 4.**
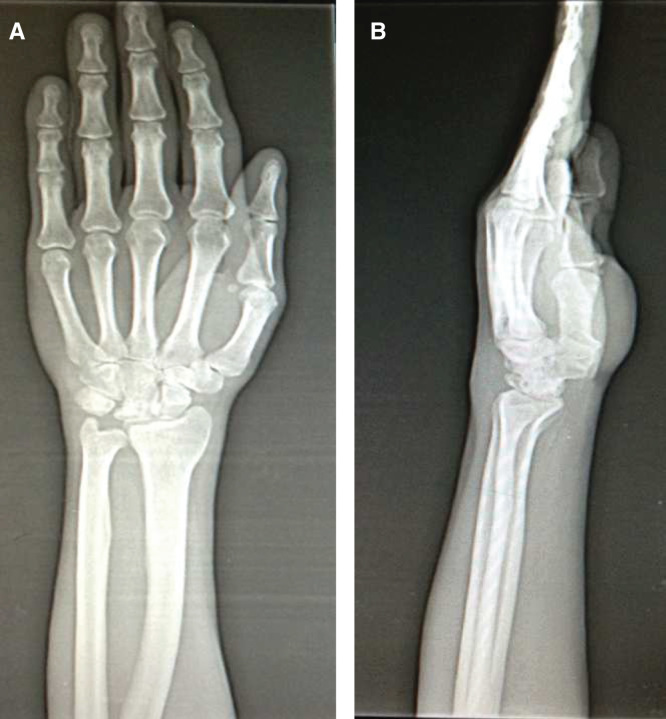
**X-rays at second year of the operations showing the lunate to be in reduced position but has been collapsed (*a* and *b*).**

A 25-year-old male patient presented to our clinic with left wrist pain radiating to the forearm, with numbness of radial three digits. He was referred by his family physician with a provisional diagnosis of CTS. EMG study had already been performed, and it showed distal motor nerve latency of 5.2 ms, sensory nerve conduction velocity of 30 m/s across the carpal tunnel and sensory nerve axon potential was not detected (normal ranges are < 4.2 ms, NR > 50 m/s and 20 µV, respectively). The median nerve entrapment was graded as moderate, as both median sensory and motor fibers were affected, and the necessity of the surgical release was informed to the patient by family physician. During our evaluation, the patient complained of pain and numbness, which began 3 months previously. Physical examination revealed hypoesthesia of the three radial digits; also, Tinel’s sign was positive, there was no motor deficit or thenar muscle atrophy. Left wrist range of movement was markedly decreased and measured as flexion 30°, extension 10°, supination 70°, and pronation 70° (range of motion of right wrist is 50°, 30°, 90°, 80°, respectively). Atypical demographic characteristics, localized volar wrist swelling, and decrease in the range of wrist joint movement were recognized and led to the initial diagnosis. Detailed history revealed that the patient had fallen down while he was climbing downstairs 5 months ago, with his left wrist forced dorsiflexion. Although he had pain and swelling in his wrist, he did not visit the hospital. In clinic plain radiographs were requested and showed isolated lunate bone dislocation, as seen in Figure 1, and CT was requested for better evaluation. Surgery was planned and performed in the sixth month post-trauma. Proximal carpectomy was one of the treatment choices but it was refused by the patient. Under general anesthesia, the flexor retinaculum was incised and the median nerve was released, then flexor tendons and median nerve were retracted, and lunate bone reduction was attempted. The lunate bone was covered by normal-looking cartilage. Failure of the anatomic reduction obligated dorsal exposure, and the dorsal capsule was incised and the lunate was successfully reduced. After anatomical reduction was achieved, fixation was applied using K-wires. A fluoroscopic control revealed normal carpal bone relationship, and both volar and dorsal capsules were repaired (Figure 2). A short arm cast was applied for 8 weeks. Three months post-operatively, all K-wires were removed, and physiotherapy was started. The patient was also evaluated 2 years postoperatively. Physical examination revealed significant improvement in symptoms and improved wrist range of motion (flexion 50°, extension 30°, supination 90°, and pronation 80°). This was comparative to range of motion of the right wrist (flexion 80°, extension 60°, supination 90°, and pronation 80°) (Figure 3). Images showed that the lunate was in the reduced position; however, there was sclerosing and shortening of lunate length (Figure 4). In spite of reduced range of motion of left wrist, the patient was able to perform all his daily activities and job demands without pain – he is a manual worker in steel industry carrying and lifting heavy objects. It is worth to mention that complete sensory recovery occurred within first month postoperatively; this was recognized by disappearance of numbness and hypoesthesia. Quick DASH score was 18.2 and Mayo wrist score was 75 (satisfactory).

## Discussion

Most cases of CTS are idiopathic; however, some local or systemic factors could contribute to CTS. The most common systemic factors are obesity, pregnancy, diabetes mellitus, rheumatoid arthritis, and osteoarthritis [1]. The local causes include inflammation, trauma, tumors, or anatomical anomalies. Several reports have been published on CTS caused by space-occupying lesions such as lipoma [2] and ganglion [3]. CTS diagnosis depends primarily on the clinical features and provocative tests. The electrodiagnostic findings are not pathognomonic; hence its role as a reference standard for diagnosis of CTS is debated. However it is essential for excluding the presence of other compression syndromes, and in postoperative follow up, since the initial findings will reveal improvement of preoperative findings. Generally patients with idiopathic CTS have several typical characteristics (female gender, increased body mass index, middle-aged, bilateral involvement). Bagatur and Zorer. stated that ∼66% of diagnosed unilateral idiopathic CTS have abnormal results of the contralateral nerve conduction test [4]. Nakamichi and Tachibana assessed the relationship between unilateral CTS and space-occupying lesion and they concluded that when the condition is unilateral and the etiology is not clear, a space-occupying lesion should be suspected [5]. When patients present with an atypical feature, the possibility of other causes of CTS such as space-occupying lesions should be considered. Therefore, more detailed history-taking, thorough physical examination, and more sophisticated investigation are required.

Isolated lunate bone dislocation is one of the uncommon injuries of the wrist and is frequently caused by high-energy trauma with the wrist dorsiflexed and in ulnar deviation (e.g., fall on an outstretched hand, and motor vehicle accidents). The lunate is more usually displaced volarly to lie within the carpal tunnel compressing the median nerve [6]. The deformity may be more subtle than expected. Patient with unreduced injuries may present very late (up to years) after the injury, although some of these cases may have good hand function with minimal pain [7]. Patients can present with pain, decreased joint range of motion, or CTS. Correct diagnosis and treatment of these injuries is the key to wrist motion restoration and pain relief. Early treatment is essential to prevent the devastating complications of chronic carpal instability and traumatic arthritis associated with missed or inappropriately treated injuries. Open reduction by both volar and dorsal approaches, with internal fixation, is the current consensus for treatment of lunate dislocation. The palmar approach is used to release the carpal transverse ligaments in addition to reduction of the lunate, whereas the dorsal approach provides good exposure of the carpus for restoring alignment and repairing scapholunate interosseous ligament, which is thought to be the key of successful long-term results [8].

Chen reported 10 patients presenting with CTS with neglected anteriorly dislocated lunate bone. Three patients were misdiagnosed as idiopathic CTS and carpal tunnel release was performed without relieve of the patients’ symptoms [9]. All 10 patients were men and all had unilateral symptoms with an average age of 32 years, which supports the possibility of secondary cause of the CTS.

Otta *et al.* has reported lunate dislocation without a history of trauma as a cause of CTS, without mentioning the demographic properties of the patient [10]. Because of improper physical examination and preoperative preparation, the lunate dislocation was recognized postoperatively, and a second operation became mandatory.

Any suspected patient with atypical demographic properties of idiopathic CTS should be carefully examined, and appropriate investigation should be obtained to exclude secondary causes.
